# *N*-(2,4-Dinitro­phen­yl)dehydro­abietyl­amine

**DOI:** 10.1107/S1600536808010490

**Published:** 2008-04-23

**Authors:** Fei Qiu, Min Hong, Dawei Jiang, Jin Zhu, Lequn Lee Huang

**Affiliations:** a School of Chemistry and Chemical Engineering, Nanjing University, Nanjing 210093, People’s Republic of China; b Department of Polymer Science and Engineering, School of Chemistry and Chemical Engineering, State Key Laboratory of Coordination Chemistry, Nanjing University, Nanjing 210093, People’s Republic of China; c Medical School, Nanjing University, Nanjing 210093, People’s Republic of China

## Abstract

In the crystal structure of the title compound, C_26_H_33_N_3_O_4_, there are two crystallographically independent mol­ecules. The two cyclohexane rings are *trans*-fused; the ring neighboring the phenyl group is in a half-chair conformation and the other is in a chair conformation. The two nitro groups and the benzene ring of the dinitro­phenyl group are almost coplanar. Intra­molecular N—H⋯O hydrogen bonds and weak inter­molecular C—H⋯O hydrogen bonds are observed.

## Related literature

For related literature, see: Baudequin *et al.* (2005 ▶); Gottstein & Cheney (1965 ▶); Jiang *et al.* (2007 ▶); Ou & Huang (2006 ▶); Pan *et al.* (2005 ▶); Patrascu *et al.* (2004 ▶).
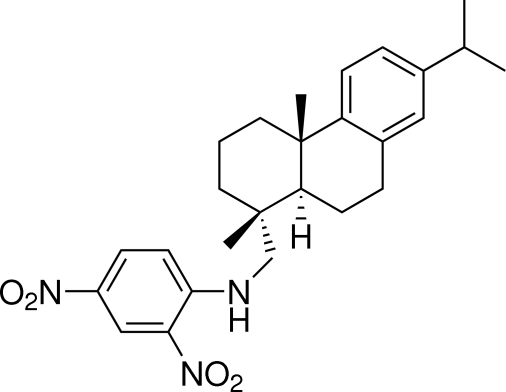


## Experimental

### 

#### Crystal data

C_26_H_33_N_3_O_4_
*M* *_r_* = 451.55
Monoclinic, 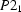

*a* = 14.119 (7) Å
*b* = 23.574 (12) Å
*c* = 7.309 (4) Å
β = 99.191 (9)°
*V* = 2402 (2) Å^3^
*Z* = 4
Mo *K*α radiation
μ = 0.09 mm^−1^
*T* = 291 (2) K
0.30 × 0.26 × 0.24 mm

#### Data collection

Bruker SMART APEX CCD diffractometer
Absorption correction: multi-scan (**SADABS**; Bruker, 2000 ▶) *T*_min_ = 0.98, *T*_max_ = 0.98
15350 measured reflections
6027 independent reflections
4715 reflections with *I* > 2σ(*I*)
*R*_int_ = 0.046

#### Refinement

*R*[*F* ^2^ > 2σ(*F* ^2^)] = 0.062
*wR*(*F* ^2^) = 0.139
*S* = 1.07
6027 reflections
609 parameters
1 restraint
H atoms treated by a mixture of independent and constrained refinement
Δρ_max_ = 0.19 e Å^−3^
Δρ_min_ = −0.19 e Å^−3^

### 

Data collection: *SMART* (Bruker, 2000 ▶); cell refinement: *SAINT* (Bruker, 2000 ▶); data reduction: *SAINT*; program(s) used to solve structure: *SHELXTL* (Sheldrick, 2008 ▶); program(s) used to refine structure: *SHELXTL*; molecular graphics: *SHELXTL*; software used to prepare material for publication: *SHELXTL*.

## Supplementary Material

Crystal structure: contains datablocks I, global. DOI: 10.1107/S1600536808010490/is2284sup1.cif

Structure factors: contains datablocks I. DOI: 10.1107/S1600536808010490/is2284Isup2.hkl

Additional supplementary materials:  crystallographic information; 3D view; checkCIF report

## Figures and Tables

**Table 1:** Hydrogen-bond geometry (Å, °)

*D*—H⋯*A*	*D*—H	H⋯*A*	*D*⋯*A*	*D*—H⋯*A*
N3—H3*A*⋯O4	0.86 (5)	1.95 (5)	2.650 (4)	137 (4)
N6—H6⋯O8	0.86 (5)	1.99 (5)	2.643 (4)	132 (4)
C10—H10*A*⋯O5^i^	0.97	2.24	3.078 (5)	144
C23—H23*B*⋯O5^i^	0.96	2.46	3.242 (5)	139
C41—H41⋯O8^ii^	0.93	2.55	3.435 (5)	159

Symmetry codes: (i) 
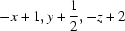
; (ii) 
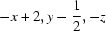
.

**Fig. 1.**
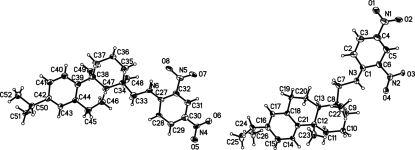
The asymmetric unit of the title compound, showing 35% probability displacement ellipsoids and the atom-numbering schemes.

**Fig. 2.**
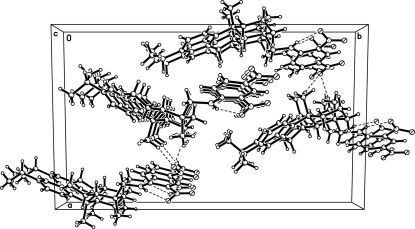
A packing diagram of the title compound, showing hydrogen bonds drawn as dashed lines.

## supplementary crystallographic information

### Comment

Chiral ionic liquids have potential applications in chiral recognition and asymmetric synthesis (Baudequin *et al.*, 2005). One way to get chiral ionic liquids is from chiral amine through Marazano's route (Patrascu *et al.*, 2004; Ou & Huang, 2006). Dehydroabietylamine which acts as a resolving agent for carboxylic acids (Gottstein & Cheney, 1965), has three chiral centres, and is inexpensive and relatively nontoxic. The title compound, (I), was an unexpected product obtained in an attempt to synthesize a chiral imidazolium ionic liquid *via* the reaction of 1-(2,4-dinitrophenyl)-3-methylimidazolium chloride with dehydroabietylamine. In this work, we describe the synthesis and crystal structure of the title compound.

As shown in Fig. 1, the asymmetric unit of (I) contains two independent molecules. Each molecule has three chiral centers. In (I), there exists four crystallographically distinct six-membered rings. Two cyclohexane rings form a *trans* ring junction with a classical chair and a half-chair conformation, and two methyl groups in axial positions (Pan *et al.*, 2005; Jiang *et al.*, 2007). Two nitro groups and the benzene ring in the substituted aryl group are almost coplanar. Intramolecular N—H···O hydrogen bonds contribute strongly to the stability of the molecular configuration (Fig. 2 and Table 1). Further analysis of the crystal packing suggests that there are some weak C—H···O interactions stabilizing the packing of (I).

### Experimental

To 1-(2,4-dinitrophenyl)-3-methylimidazolium chloride (3.0 g, 10.5 mmol) suspended in n-butanol (50 ml) was added a solution of dehydroabietylamine (3.3 g, 11.5 mmol) in n-butanol (10 ml) and the mixture was refluxed for 24 h. Removal of solvent under reduced pressure left a residue, which was further purified by column chromatography (n-hexane/ethyl acetate as eluent) and then recrystallized from methanol to give the title compound (yield 35.5%, m.p. 408–409 K).

### Refinement

H atoms attached to C atoms were positioned geometrically (C—H = 0.93–0.98 Å) and were refined as riding, with *U*_iso_(H) = 1.2*U*_eq_(C) or 1.5*U*_eq_(methyl C). The coordinates of the H atoms bonded to N were refined with *U*_iso_(H) = 1.2*U*_eq_(N), giving the N—H distance of 0.86 (5) Å. In the absence of significant anomalous scattering effects, Friedel pairs have been merged.

### Crystal data

**Table:**

C_26_H_33_N_3_O_4_	*F*_000_ = 968
*M**_r_* = 451.55	*D*_x_ = 1.249 Mg m^−^^3^
Monoclinic, *P*2_1_	Mo *K*α radiation λ = 0.71073 Å
Hall symbol: P 2yb	Cell parameters from 1357 reflections
*a* = 14.119 (7) Å	θ = 2.9–18.1º
*b* = 23.574 (12) Å	µ = 0.09 mm^−^^1^
*c* = 7.309 (4) Å	*T* = 291 (2) K
β = 99.191 (9)º	Block, light-yellow
*V* = 2402 (2) Å^3^	0.30 × 0.26 × 0.24 mm
*Z* = 4	

### Data collection

**Table:**

Bruker SMART APEX CCD diffractometer	6027 independent reflections
Radiation source: sealed tube	4715 reflections with *I* > 2σ(*I*)
Monochromator: graphite	*R*_int_ = 0.046
*T* = 291(2) K	θ_max_ = 28.6º
φ and ω scans	θ_min_ = 1.7º
Absorption correction: multi-scan(*SADABS*; Bruker, 2000)	*h* = −16→18
*T*_min_ = 0.98, *T*_max_ = 0.98	*k* = −31→31
15350 measured reflections	*l* = −9→9

### Refinement

**Table:**

Refinement on *F*^2^	Secondary atom site location: difference Fourier map
Least-squares matrix: full	Hydrogen site location: inferred from neighbouring sites
*R*[*F*^2^ > 2σ(*F*^2^)] = 0.062	H atoms treated by a mixture of independent and constrained refinement
*wR*(*F*^2^) = 0.139	*w* = 1/[σ^2^(*F*_o_^2^) + (0.07*P*)^2^] where *P* = (*F*_o_^2^ + 2*F*_c_^2^)/3
*S* = 1.07	(Δ/σ)_max_ < 0.001
6027 reflections	Δρ_max_ = 0.19 e Å^−^^3^
609 parameters	Δρ_min_ = −0.19 e Å^−^^3^
1 restraint	Extinction correction: none
Primary atom site location: structure-invariant direct methods	

### Special details

**Table:**

Geometry. All e.s.d.'s (except the e.s.d. in the dihedral angle between two l.s. planes) are estimated using the full covariance matrix. The cell e.s.d.'s are taken into account individually in the estimation of e.s.d.'s in distances, angles and torsion angles; correlations between e.s.d.'s in cell parameters are only used when they are defined by crystal symmetry. An approximate (isotropic) treatment of cell e.s.d.'s is used for estimating e.s.d.'s involving l.s. planes.
Refinement. Refinement of *F*^2^ against ALL reflections. The weighted *R*-factor *wR* and goodness of fit *S* are based on *F*^2^, conventional *R*-factors *R* are based on *F*, with *F* set to zero for negative *F*^2^. The threshold expression of *F*^2^ > σ(*F*^2^) is used only for calculating *R*-factors(gt) *etc*. and is not relevant to the choice of reflections for refinement. *R*-factors based on *F*^2^ are statistically about twice as large as those based on *F*, and *R*- factors based on ALL data will be even larger.

### Fractional atomic coordinates and isotropic or equivalent isotropic displacement parameters (Å^2^)

**Table:**

	*x*	*y*	*z*	*U*_iso_*/*U*_eq_	
C1	0.6051 (3)	1.03496 (15)	0.3284 (6)	0.0432 (8)	
C2	0.6497 (3)	1.01296 (18)	0.1815 (5)	0.0433 (8)	
H2	0.6574	0.9738	0.1763	0.052*	
C3	0.6826 (3)	1.0453 (2)	0.0451 (6)	0.0539 (10)	
H3	0.7101	1.0292	−0.0501	0.065*	
C4	0.6711 (3)	1.1052 (2)	0.0627 (7)	0.0562 (11)	
C5	0.6347 (3)	1.12950 (17)	0.2041 (6)	0.0481 (9)	
H5	0.6308	1.1688	0.2131	0.058*	
C6	0.6036 (3)	1.09476 (15)	0.3349 (5)	0.0419 (8)	
C7	0.5823 (3)	0.93895 (15)	0.4645 (6)	0.0462 (9)	
H7A	0.6455	0.9286	0.5285	0.055*	
H7B	0.5737	0.9215	0.3428	0.055*	
C8	0.5050 (2)	0.91540 (12)	0.5747 (5)	0.0331 (7)	
C9	0.5037 (3)	0.94785 (13)	0.7600 (5)	0.0386 (8)	
H9A	0.5694	0.9527	0.8211	0.046*	
H9B	0.4773	0.9854	0.7309	0.046*	
C10	0.4483 (3)	0.92050 (15)	0.8925 (6)	0.0447 (9)	
H10A	0.3817	0.9166	0.8359	0.054*	
H10B	0.4509	0.9439	1.0024	0.054*	
C11	0.4910 (3)	0.86223 (14)	0.9458 (5)	0.0415 (8)	
H11A	0.4554	0.8445	1.0336	0.050*	
H11B	0.5570	0.8666	1.0057	0.050*	
C12	0.4876 (3)	0.82217 (15)	0.7685 (5)	0.0381 (7)	
C13	0.5412 (3)	0.76731 (14)	0.8357 (5)	0.0357 (7)	
C14	0.5280 (2)	0.74107 (13)	0.9926 (5)	0.0344 (7)	
H14	0.4851	0.7570	1.0623	0.041*	
C15	0.5761 (3)	0.69012 (16)	1.0579 (5)	0.0459 (8)	
H15	0.5650	0.6737	1.1683	0.055*	
C16	0.6404 (3)	0.66476 (14)	0.9544 (5)	0.0398 (8)	
C17	0.6516 (3)	0.69130 (14)	0.7860 (5)	0.0407 (8)	
H17	0.6923	0.6752	0.7122	0.049*	
C18	0.6025 (2)	0.74134 (14)	0.7281 (5)	0.0352 (7)	
C19	0.6249 (3)	0.76646 (14)	0.5425 (5)	0.0390 (8)	
H19A	0.6892	0.7822	0.5620	0.047*	
H19B	0.6224	0.7365	0.4510	0.047*	
C20	0.5532 (3)	0.81258 (14)	0.4705 (5)	0.0383 (8)	
H20A	0.4914	0.7958	0.4236	0.046*	
H20B	0.5752	0.8331	0.3701	0.046*	
C21	0.5437 (3)	0.85369 (13)	0.6321 (5)	0.0331 (7)	
H21	0.6089	0.8590	0.6994	0.040*	
C22	0.4086 (3)	0.91669 (18)	0.4435 (6)	0.0513 (10)	
H22A	0.3573	0.9200	0.5147	0.077*	
H22B	0.4010	0.8823	0.3723	0.077*	
H22C	0.4072	0.9486	0.3615	0.077*	
C23	0.3848 (3)	0.80579 (14)	0.7005 (5)	0.0421 (8)	
H23A	0.3797	0.7896	0.5788	0.063*	
H23B	0.3448	0.8389	0.6957	0.063*	
H23C	0.3644	0.7785	0.7836	0.063*	
C24	0.6938 (3)	0.61086 (15)	1.0142 (6)	0.0420 (8)	
H24	0.7451	0.6057	0.9399	0.050*	
C25	0.6235 (3)	0.56052 (16)	0.9783 (6)	0.0513 (10)	
H25A	0.6583	0.5268	0.9571	0.077*	
H25B	0.5764	0.5684	0.8713	0.077*	
H25C	0.5921	0.5551	1.0842	0.077*	
C26	0.7371 (3)	0.61064 (15)	1.2165 (6)	0.0462 (9)	
H26A	0.6879	0.6031	1.2901	0.069*	
H26B	0.7655	0.6470	1.2499	0.069*	
H26C	0.7854	0.5817	1.2386	0.069*	
C27	0.8660 (3)	0.30147 (16)	0.5585 (5)	0.0461 (9)	
C28	0.8144 (2)	0.28052 (15)	0.6931 (5)	0.0364 (7)	
H28	0.7932	0.2431	0.6848	0.044*	
C29	0.7940 (3)	0.31439 (17)	0.8397 (6)	0.0476 (9)	
H29	0.7646	0.2991	0.9338	0.057*	
C30	0.8189 (3)	0.37176 (18)	0.8405 (5)	0.0461 (9)	
C31	0.8630 (3)	0.39573 (17)	0.7035 (6)	0.0481 (9)	
H31	0.8753	0.4345	0.7041	0.058*	
C32	0.8884 (3)	0.36200 (17)	0.5670 (6)	0.0496 (10)	
C33	0.8782 (3)	0.20752 (18)	0.4190 (7)	0.0526 (10)	
H33A	0.8187	0.1987	0.3378	0.063*	
H33B	0.8737	0.1928	0.5413	0.063*	
C34	0.9638 (2)	0.17850 (16)	0.3441 (5)	0.0394 (8)	
C35	0.9616 (3)	0.2017 (2)	0.1400 (6)	0.0533 (10)	
H35A	0.8959	0.2009	0.0759	0.064*	
H35B	0.9825	0.2409	0.1467	0.064*	
C36	1.0223 (3)	0.16940 (16)	0.0306 (6)	0.0451 (9)	
H36A	1.0886	0.1716	0.0908	0.054*	
H36B	1.0181	0.1864	−0.0913	0.054*	
C37	0.9912 (3)	0.10552 (17)	0.0095 (6)	0.0451 (9)	
H37A	0.9266	0.1031	−0.0593	0.054*	
H37B	1.0338	0.0855	−0.0601	0.054*	
C38	0.9944 (3)	0.07674 (16)	0.2038 (5)	0.0410 (8)	
C39	0.9476 (3)	0.01943 (17)	0.1764 (6)	0.0462 (9)	
C40	0.9525 (3)	−0.01538 (18)	0.0191 (6)	0.0487 (9)	
H40	0.9807	−0.0001	−0.0765	0.058*	
C41	0.9189 (3)	−0.06933 (18)	−0.0008 (6)	0.0527 (10)	
H41	0.9299	−0.0920	−0.0995	0.063*	
C42	0.8665 (3)	−0.08959 (17)	0.1350 (6)	0.0495 (9)	
C43	0.8643 (3)	−0.05987 (15)	0.2910 (6)	0.0469 (9)	
H43	0.8387	−0.0768	0.3871	0.056*	
C44	0.8990 (3)	−0.00449 (16)	0.3140 (6)	0.0454 (9)	
C45	0.8833 (3)	0.02884 (19)	0.4823 (5)	0.0504 (9)	
H45A	0.8157	0.0382	0.4710	0.060*	
H45B	0.8995	0.0050	0.5910	0.060*	
C46	0.9406 (3)	0.08257 (18)	0.5118 (5)	0.0472 (9)	
H46A	1.0063	0.0738	0.5659	0.057*	
H46B	0.9137	0.1070	0.5971	0.057*	
C47	0.9395 (3)	0.11368 (17)	0.3245 (5)	0.0414 (8)	
H47	0.8725	0.1122	0.2635	0.050*	
C48	1.0574 (3)	0.19114 (17)	0.4684 (6)	0.0478 (9)	
H48A	1.0632	0.2313	0.4892	0.072*	
H48B	1.1098	0.1781	0.4102	0.072*	
H48C	1.0589	0.1720	0.5847	0.072*	
C49	1.1003 (3)	0.06585 (17)	0.2926 (6)	0.0477 (9)	
H49A	1.1022	0.0490	0.4127	0.071*	
H49B	1.1346	0.1012	0.3049	0.071*	
H49C	1.1297	0.0407	0.2150	0.071*	
C50	0.8210 (3)	−0.15026 (17)	0.1030 (6)	0.0457 (9)	
H50	0.7520	−0.1475	0.1065	0.055*	
C51	0.8665 (3)	−0.18691 (18)	0.2626 (6)	0.0485 (9)	
H51A	0.8173	−0.2052	0.3177	0.073*	
H51B	0.9050	−0.1637	0.3537	0.073*	
H51C	0.9063	−0.2151	0.2180	0.073*	
C52	0.8352 (3)	−0.18168 (19)	−0.0710 (5)	0.0524 (10)	
H52A	0.8988	−0.1972	−0.0552	0.079*	
H52B	0.8266	−0.1559	−0.1740	0.079*	
H52C	0.7891	−0.2118	−0.0941	0.079*	
N1	0.7052 (2)	1.13979 (16)	−0.0758 (5)	0.0526 (9)	
N2	0.5607 (2)	1.12477 (13)	0.4765 (5)	0.0467 (8)	
N3	0.5767 (3)	1.00406 (13)	0.4424 (5)	0.0487 (8)	
H3A	0.549 (3)	1.021 (2)	0.523 (7)	0.058*	
N4	0.7997 (2)	0.40656 (16)	0.9895 (6)	0.0549 (9)	
N5	0.9317 (3)	0.39006 (16)	0.4270 (6)	0.0568 (9)	
N6	0.8912 (2)	0.26788 (15)	0.4290 (5)	0.0478 (8)	
H6	0.918 (3)	0.284 (2)	0.344 (7)	0.057*	
O1	0.7368 (2)	1.11758 (13)	−0.2038 (4)	0.0527 (7)	
O2	0.70301 (19)	1.19145 (13)	−0.0571 (4)	0.0495 (7)	
O3	0.57835 (17)	1.17599 (9)	0.4971 (4)	0.0389 (5)	
O4	0.5187 (2)	1.09936 (10)	0.5818 (4)	0.0450 (6)	
O5	0.7625 (2)	0.38555 (12)	1.1176 (4)	0.0506 (7)	
O6	0.8212 (2)	0.45609 (12)	0.9977 (4)	0.0512 (7)	
O7	0.93068 (18)	0.44089 (12)	0.4172 (4)	0.0479 (6)	
O8	0.9785 (2)	0.35937 (12)	0.3293 (4)	0.0494 (7)	

### Atomic displacement parameters (Å^2^)

**Table:**

	*U*^11^	*U*^22^	*U*^33^	*U*^12^	*U*^13^	*U*^23^
C1	0.055 (2)	0.0282 (17)	0.048 (2)	−0.0011 (15)	0.0145 (17)	0.0057 (15)
C2	0.0429 (18)	0.049 (2)	0.0401 (19)	−0.0011 (16)	0.0125 (16)	0.0006 (16)
C3	0.059 (2)	0.059 (3)	0.045 (2)	−0.004 (2)	0.016 (2)	0.0064 (19)
C4	0.051 (2)	0.062 (3)	0.059 (3)	0.0028 (19)	0.0181 (19)	0.022 (2)
C5	0.0405 (19)	0.0402 (19)	0.063 (3)	0.0012 (15)	0.0062 (18)	0.0205 (18)
C6	0.060 (2)	0.0285 (16)	0.0371 (18)	0.0034 (15)	0.0081 (16)	0.0037 (14)
C7	0.065 (2)	0.0340 (18)	0.044 (2)	−0.0035 (17)	0.0238 (18)	0.0093 (15)
C8	0.0305 (15)	0.0190 (13)	0.0514 (19)	0.0003 (11)	0.0117 (14)	−0.0050 (13)
C9	0.0415 (16)	0.0232 (14)	0.057 (2)	0.0065 (13)	0.0248 (16)	−0.0095 (14)
C10	0.056 (2)	0.0351 (18)	0.049 (2)	0.0085 (16)	0.0267 (18)	0.0059 (15)
C11	0.056 (2)	0.0302 (17)	0.0373 (18)	0.0018 (15)	0.0051 (16)	−0.0096 (14)
C12	0.0418 (18)	0.0327 (17)	0.0418 (19)	−0.0009 (14)	0.0127 (15)	−0.0057 (14)
C13	0.0452 (18)	0.0285 (15)	0.0355 (16)	−0.0006 (14)	0.0131 (14)	−0.0032 (13)
C14	0.0433 (17)	0.0211 (13)	0.0437 (18)	−0.0080 (13)	0.0219 (14)	−0.0025 (13)
C15	0.059 (2)	0.0371 (19)	0.042 (2)	−0.0016 (17)	0.0098 (17)	0.0057 (16)
C16	0.0444 (18)	0.0261 (16)	0.046 (2)	0.0035 (14)	−0.0019 (15)	0.0028 (14)
C17	0.054 (2)	0.0240 (15)	0.0467 (19)	−0.0027 (14)	0.0147 (16)	0.0017 (14)
C18	0.0411 (17)	0.0337 (16)	0.0341 (16)	0.0100 (14)	0.0159 (14)	0.0021 (13)
C19	0.057 (2)	0.0338 (17)	0.0321 (16)	0.0245 (15)	0.0254 (15)	0.0071 (13)
C20	0.0441 (18)	0.0261 (15)	0.049 (2)	0.0134 (13)	0.0197 (16)	−0.0029 (14)
C21	0.0447 (18)	0.0207 (13)	0.0336 (17)	0.0115 (12)	0.0053 (14)	0.0012 (12)
C22	0.046 (2)	0.042 (2)	0.059 (2)	0.0082 (16)	−0.0129 (18)	0.0162 (18)
C23	0.052 (2)	0.0274 (16)	0.0436 (19)	−0.0006 (14)	−0.0014 (16)	−0.0077 (14)
C24	0.0447 (19)	0.0308 (16)	0.050 (2)	−0.0030 (14)	0.0054 (16)	0.0094 (15)
C25	0.059 (2)	0.0331 (19)	0.055 (2)	0.0007 (16)	−0.0121 (19)	−0.0160 (16)
C26	0.050 (2)	0.0294 (17)	0.052 (2)	0.0095 (14)	−0.0156 (17)	−0.0079 (15)
C27	0.060 (2)	0.040 (2)	0.043 (2)	−0.0045 (16)	0.0252 (18)	0.0103 (16)
C28	0.0326 (15)	0.0414 (18)	0.0382 (17)	−0.0001 (13)	0.0152 (13)	0.0158 (14)
C29	0.048 (2)	0.047 (2)	0.053 (2)	0.0011 (17)	0.0230 (18)	0.0061 (17)
C30	0.045 (2)	0.053 (2)	0.039 (2)	0.0013 (16)	0.0026 (16)	0.0023 (16)
C31	0.051 (2)	0.044 (2)	0.053 (2)	0.0036 (17)	0.0191 (18)	0.0070 (17)
C32	0.052 (2)	0.044 (2)	0.057 (2)	0.0019 (17)	0.0231 (19)	0.0163 (18)
C33	0.049 (2)	0.053 (2)	0.059 (3)	0.0070 (18)	0.020 (2)	0.0074 (19)
C34	0.0322 (15)	0.049 (2)	0.0383 (18)	−0.0087 (14)	0.0087 (14)	−0.0041 (15)
C35	0.057 (2)	0.067 (3)	0.038 (2)	0.013 (2)	0.0137 (17)	0.0020 (18)
C36	0.0416 (19)	0.047 (2)	0.052 (2)	−0.0066 (16)	0.0224 (17)	0.0010 (17)
C37	0.050 (2)	0.050 (2)	0.0422 (19)	−0.0061 (16)	0.0277 (16)	0.0090 (16)
C38	0.054 (2)	0.0417 (19)	0.0286 (16)	0.0002 (16)	0.0112 (15)	0.0004 (14)
C39	0.049 (2)	0.042 (2)	0.050 (2)	0.0074 (16)	0.0171 (17)	0.0019 (16)
C40	0.046 (2)	0.053 (2)	0.050 (2)	−0.0102 (17)	0.0156 (18)	−0.0104 (18)
C41	0.052 (2)	0.053 (2)	0.060 (3)	−0.0086 (18)	0.030 (2)	−0.0151 (19)
C42	0.053 (2)	0.046 (2)	0.056 (2)	0.0008 (17)	0.0279 (18)	−0.0146 (18)
C43	0.060 (2)	0.0311 (18)	0.055 (2)	0.0016 (16)	0.0264 (19)	−0.0056 (16)
C44	0.0364 (17)	0.047 (2)	0.057 (2)	−0.0018 (15)	0.0211 (17)	−0.0112 (17)
C45	0.056 (2)	0.060 (2)	0.0368 (19)	−0.0081 (19)	0.0127 (17)	−0.0130 (17)
C46	0.060 (2)	0.058 (2)	0.0237 (16)	−0.0101 (18)	0.0073 (16)	−0.0009 (15)
C47	0.0413 (18)	0.053 (2)	0.0330 (17)	−0.0014 (15)	0.0147 (14)	−0.0052 (15)
C48	0.0393 (18)	0.043 (2)	0.058 (2)	0.0094 (15)	−0.0001 (17)	0.0002 (18)
C49	0.048 (2)	0.046 (2)	0.056 (2)	0.0143 (17)	0.0266 (18)	0.0156 (17)
C50	0.046 (2)	0.044 (2)	0.050 (2)	−0.0041 (16)	0.0168 (17)	−0.0169 (17)
C51	0.049 (2)	0.052 (2)	0.052 (2)	0.0156 (17)	0.0310 (18)	−0.0003 (17)
C52	0.069 (3)	0.054 (2)	0.0344 (19)	−0.013 (2)	0.0110 (18)	−0.0182 (17)
N1	0.0447 (18)	0.062 (2)	0.051 (2)	−0.0056 (16)	0.0073 (15)	0.0190 (18)
N2	0.0492 (18)	0.0325 (16)	0.061 (2)	0.0058 (13)	0.0167 (16)	−0.0006 (14)
N3	0.060 (2)	0.0323 (15)	0.059 (2)	0.0134 (14)	0.0258 (17)	0.0106 (14)
N4	0.0407 (16)	0.059 (2)	0.074 (2)	−0.0129 (15)	0.0368 (17)	−0.0182 (18)
N5	0.060 (2)	0.052 (2)	0.065 (2)	−0.0081 (17)	0.0302 (18)	0.0090 (18)
N6	0.0416 (16)	0.055 (2)	0.055 (2)	0.0091 (14)	0.0337 (15)	0.0108 (16)
O1	0.0482 (15)	0.0534 (17)	0.0579 (18)	0.0120 (13)	0.0132 (13)	0.0199 (14)
O2	0.0402 (14)	0.0585 (17)	0.0540 (16)	−0.0084 (12)	0.0203 (12)	0.0111 (13)
O3	0.0461 (12)	0.0225 (11)	0.0516 (14)	0.0126 (9)	0.0185 (11)	0.0055 (10)
O4	0.0571 (16)	0.0349 (13)	0.0460 (14)	0.0099 (12)	0.0175 (12)	0.0055 (11)
O5	0.0531 (15)	0.0542 (16)	0.0460 (15)	−0.0260 (12)	0.0124 (12)	−0.0079 (12)
O6	0.0542 (16)	0.0463 (16)	0.0557 (17)	−0.0035 (12)	0.0166 (13)	−0.0040 (13)
O7	0.0435 (13)	0.0539 (17)	0.0458 (15)	−0.0245 (12)	0.0057 (11)	0.0123 (12)
O8	0.0535 (16)	0.0477 (15)	0.0511 (16)	−0.0099 (12)	0.0208 (13)	0.0168 (12)

### Geometric parameters (Å, °)

**Table:**

C1—N3	1.222 (5)	C29—C30	1.397 (6)
C1—C6	1.411 (5)	C29—H29	0.9300
C1—C2	1.426 (5)	C30—C31	1.381 (5)
C2—C3	1.392 (5)	C30—N4	1.424 (5)
C2—H2	0.9300	C31—C32	1.367 (6)
C3—C4	1.429 (7)	C31—H31	0.9300
C3—H3	0.9300	C32—N5	1.435 (5)
C4—C5	1.352 (6)	C33—N6	1.435 (6)
C4—N1	1.441 (5)	C33—C34	1.562 (5)
C5—C6	1.383 (5)	C33—H33A	0.9700
C5—H5	0.9300	C33—H33B	0.9700
C6—N2	1.462 (5)	C34—C48	1.510 (5)
C7—N3	1.544 (5)	C34—C47	1.568 (5)
C7—C8	1.558 (5)	C34—C35	1.584 (5)
C7—H7A	0.9700	C35—C36	1.474 (5)
C7—H7B	0.9700	C35—H35A	0.9700
C8—C22	1.535 (5)	C35—H35B	0.9700
C8—C9	1.558 (5)	C36—C37	1.569 (5)
C8—C21	1.587 (4)	C36—H36A	0.9700
C9—C10	1.486 (5)	C36—H36B	0.9700
C9—H9A	0.9700	C37—C38	1.568 (5)
C9—H9B	0.9700	C37—H37A	0.9700
C10—C11	1.525 (5)	C37—H37B	0.9700
C10—H10A	0.9700	C38—C39	1.503 (6)
C10—H10B	0.9700	C38—C47	1.535 (5)
C11—C12	1.598 (5)	C38—C49	1.554 (6)
C11—H11A	0.9700	C39—C44	1.421 (5)
C11—H11B	0.9700	C39—C40	1.423 (6)
C12—C23	1.508 (5)	C40—C41	1.357 (6)
C12—C13	1.539 (5)	C40—H40	0.9300
C12—C21	1.557 (5)	C41—C42	1.413 (5)
C13—C14	1.342 (5)	C41—H41	0.9300
C13—C18	1.400 (4)	C42—C43	1.343 (5)
C14—C15	1.424 (5)	C42—C50	1.570 (6)
C14—H14	0.9300	C43—C44	1.395 (5)
C15—C16	1.405 (5)	C43—H43	0.9300
C15—H15	0.9300	C44—C45	1.506 (5)
C16—C17	1.412 (5)	C45—C46	1.500 (6)
C16—C24	1.506 (5)	C45—H45A	0.9700
C17—C18	1.399 (5)	C45—H45B	0.9700
C17—H17	0.9300	C46—C47	1.551 (5)
C18—C19	1.558 (4)	C46—H46A	0.9700
C19—C20	1.521 (4)	C46—H46B	0.9700
C19—H19A	0.9700	C47—H47	0.9800
C19—H19B	0.9700	C48—H48A	0.9600
C20—C21	1.550 (4)	C48—H48B	0.9600
C20—H20A	0.9700	C48—H48C	0.9600
C20—H20B	0.9700	C49—H49A	0.9600
C21—H21	0.9800	C49—H49B	0.9600
C22—H22A	0.9600	C49—H49C	0.9600
C22—H22B	0.9600	C50—C51	1.511 (6)
C22—H22C	0.9600	C50—C52	1.512 (5)
C23—H23A	0.9600	C50—H50	0.9800
C23—H23B	0.9600	C51—H51A	0.9600
C23—H23C	0.9600	C51—H51B	0.9600
C24—C26	1.507 (5)	C51—H51C	0.9600
C24—C25	1.542 (5)	C52—H52A	0.9600
C24—H24	0.9800	C52—H52B	0.9600
C25—H25A	0.9600	C52—H52C	0.9600
C25—H25B	0.9600	N1—O1	1.217 (5)
C25—H25C	0.9600	N1—O2	1.226 (5)
C26—H26A	0.9600	N2—O4	1.204 (4)
C26—H26B	0.9600	N2—O3	1.237 (4)
C26—H26C	0.9600	N3—H3A	0.86 (5)
C27—N6	1.326 (5)	N4—O6	1.206 (4)
C27—C28	1.404 (4)	N4—O5	1.247 (4)
C27—C32	1.461 (6)	N5—O7	1.200 (5)
C28—C29	1.403 (5)	N5—O8	1.273 (5)
C28—H28	0.9300	N6—H6	0.86 (5)
			
N3—C1—C6	124.4 (4)	C31—C30—C29	122.4 (4)
N3—C1—C2	121.9 (4)	C31—C30—N4	118.8 (4)
C6—C1—C2	113.5 (3)	C29—C30—N4	118.8 (4)
C3—C2—C1	125.3 (4)	C32—C31—C30	119.5 (4)
C3—C2—H2	117.4	C32—C31—H31	120.3
C1—C2—H2	117.4	C30—C31—H31	120.3
C2—C3—C4	114.9 (4)	C31—C32—N5	116.3 (4)
C2—C3—H3	122.6	C31—C32—C27	121.3 (3)
C4—C3—H3	122.6	N5—C32—C27	122.3 (4)
C5—C4—C3	123.4 (4)	N6—C33—C34	110.6 (3)
C5—C4—N1	120.5 (4)	N6—C33—H33A	109.5
C3—C4—N1	116.1 (4)	C34—C33—H33A	109.5
C4—C5—C6	118.6 (4)	N6—C33—H33B	109.5
C4—C5—H5	120.7	C34—C33—H33B	109.5
C6—C5—H5	120.7	H33A—C33—H33B	108.1
C5—C6—C1	124.1 (4)	C48—C34—C33	110.8 (3)
C5—C6—N2	114.6 (3)	C48—C34—C47	113.9 (3)
C1—C6—N2	121.2 (3)	C33—C34—C47	106.8 (3)
N3—C7—C8	112.3 (3)	C48—C34—C35	112.5 (3)
N3—C7—H7A	109.1	C33—C34—C35	106.2 (3)
C8—C7—H7A	109.1	C47—C34—C35	106.2 (3)
N3—C7—H7B	109.1	C36—C35—C34	114.0 (3)
C8—C7—H7B	109.1	C36—C35—H35A	108.8
H7A—C7—H7B	107.9	C34—C35—H35A	108.8
C22—C8—C9	113.6 (3)	C36—C35—H35B	108.8
C22—C8—C7	107.1 (3)	C34—C35—H35B	108.8
C9—C8—C7	112.4 (3)	H35A—C35—H35B	107.6
C22—C8—C21	114.6 (3)	C35—C36—C37	111.8 (3)
C9—C8—C21	105.8 (3)	C35—C36—H36A	109.3
C7—C8—C21	102.9 (3)	C37—C36—H36A	109.3
C10—C9—C8	115.7 (3)	C35—C36—H36B	109.3
C10—C9—H9A	108.3	C37—C36—H36B	109.3
C8—C9—H9A	108.3	H36A—C36—H36B	107.9
C10—C9—H9B	108.3	C38—C37—C36	111.0 (3)
C8—C9—H9B	108.3	C38—C37—H37A	109.4
H9A—C9—H9B	107.4	C36—C37—H37A	109.4
C9—C10—C11	109.3 (3)	C38—C37—H37B	109.4
C9—C10—H10A	109.8	C36—C37—H37B	109.4
C11—C10—H10A	109.8	H37A—C37—H37B	108.0
C9—C10—H10B	109.8	C39—C38—C47	109.5 (3)
C11—C10—H10B	109.8	C39—C38—C49	106.3 (3)
H10A—C10—H10B	108.3	C47—C38—C49	113.3 (3)
C10—C11—C12	111.4 (3)	C39—C38—C37	108.6 (3)
C10—C11—H11A	109.3	C47—C38—C37	109.3 (3)
C12—C11—H11A	109.3	C49—C38—C37	109.7 (3)
C10—C11—H11B	109.3	C44—C39—C40	115.7 (4)
C12—C11—H11B	109.3	C44—C39—C38	120.9 (3)
H11A—C11—H11B	108.0	C40—C39—C38	123.3 (3)
C23—C12—C13	106.8 (3)	C41—C40—C39	124.4 (4)
C23—C12—C21	118.2 (3)	C41—C40—H40	117.8
C13—C12—C21	109.3 (3)	C39—C40—H40	117.8
C23—C12—C11	108.7 (3)	C40—C41—C42	117.1 (4)
C13—C12—C11	106.7 (3)	C40—C41—H41	121.5
C21—C12—C11	106.5 (3)	C42—C41—H41	121.5
C14—C13—C18	117.5 (3)	C43—C42—C41	120.4 (4)
C14—C13—C12	122.1 (3)	C43—C42—C50	122.5 (3)
C18—C13—C12	120.3 (3)	C41—C42—C50	116.8 (3)
C13—C14—C15	123.5 (3)	C42—C43—C44	122.4 (4)
C13—C14—H14	118.3	C42—C43—H43	118.8
C15—C14—H14	118.3	C44—C43—H43	118.8
C16—C15—C14	119.5 (3)	C43—C44—C39	119.1 (3)
C16—C15—H15	120.2	C43—C44—C45	119.5 (3)
C14—C15—H15	120.2	C39—C44—C45	121.4 (4)
C15—C16—C17	116.9 (3)	C46—C45—C44	114.3 (3)
C15—C16—C24	122.6 (3)	C46—C45—H45A	108.7
C17—C16—C24	120.4 (3)	C44—C45—H45A	108.7
C18—C17—C16	121.2 (3)	C46—C45—H45B	108.7
C18—C17—H17	119.4	C44—C45—H45B	108.7
C16—C17—H17	119.4	H45A—C45—H45B	107.6
C17—C18—C13	121.3 (3)	C45—C46—C47	110.1 (3)
C17—C18—C19	115.1 (3)	C45—C46—H46A	109.6
C13—C18—C19	123.6 (3)	C47—C46—H46A	109.6
C20—C19—C18	110.9 (3)	C45—C46—H46B	109.6
C20—C19—H19A	109.5	C47—C46—H46B	109.6
C18—C19—H19A	109.5	H46A—C46—H46B	108.2
C20—C19—H19B	109.5	C38—C47—C46	107.7 (3)
C18—C19—H19B	109.5	C38—C47—C34	118.8 (3)
H19A—C19—H19B	108.1	C46—C47—C34	114.1 (3)
C19—C20—C21	108.5 (3)	C38—C47—H47	105.0
C19—C20—H20A	110.0	C46—C47—H47	105.0
C21—C20—H20A	110.0	C34—C47—H47	105.0
C19—C20—H20B	110.0	C34—C48—H48A	109.5
C21—C20—H20B	110.0	C34—C48—H48B	109.5
H20A—C20—H20B	108.4	H48A—C48—H48B	109.5
C20—C21—C12	107.7 (3)	C34—C48—H48C	109.5
C20—C21—C8	116.1 (3)	H48A—C48—H48C	109.5
C12—C21—C8	114.7 (3)	H48B—C48—H48C	109.5
C20—C21—H21	105.8	C38—C49—H49A	109.5
C12—C21—H21	105.8	C38—C49—H49B	109.5
C8—C21—H21	105.8	H49A—C49—H49B	109.5
C8—C22—H22A	109.5	C38—C49—H49C	109.5
C8—C22—H22B	109.5	H49A—C49—H49C	109.5
H22A—C22—H22B	109.5	H49B—C49—H49C	109.5
C8—C22—H22C	109.5	C51—C50—C52	105.8 (3)
H22A—C22—H22C	109.5	C51—C50—C42	107.0 (3)
H22B—C22—H22C	109.5	C52—C50—C42	117.7 (3)
C12—C23—H23A	109.5	C51—C50—H50	108.7
C12—C23—H23B	109.5	C52—C50—H50	108.7
H23A—C23—H23B	109.5	C42—C50—H50	108.7
C12—C23—H23C	109.5	C50—C51—H51A	109.5
H23A—C23—H23C	109.5	C50—C51—H51B	109.5
H23B—C23—H23C	109.5	H51A—C51—H51B	109.5
C16—C24—C26	113.2 (3)	C50—C51—H51C	109.5
C16—C24—C25	108.8 (3)	H51A—C51—H51C	109.5
C26—C24—C25	108.3 (3)	H51B—C51—H51C	109.5
C16—C24—H24	108.9	C50—C52—H52A	109.5
C26—C24—H24	108.9	C50—C52—H52B	109.5
C25—C24—H24	108.9	H52A—C52—H52B	109.5
C24—C25—H25A	109.5	C50—C52—H52C	109.5
C24—C25—H25B	109.5	H52A—C52—H52C	109.5
H25A—C25—H25B	109.5	H52B—C52—H52C	109.5
C24—C25—H25C	109.5	O1—N1—O2	122.1 (3)
H25A—C25—H25C	109.5	O1—N1—C4	120.1 (4)
H25B—C25—H25C	109.5	O2—N1—C4	117.8 (4)
C24—C26—H26A	109.5	O4—N2—O3	121.2 (3)
C24—C26—H26B	109.5	O4—N2—C6	120.9 (3)
H26A—C26—H26B	109.5	O3—N2—C6	117.4 (3)
C24—C26—H26C	109.5	C1—N3—C7	130.3 (3)
H26A—C26—H26C	109.5	C1—N3—H3A	115 (3)
H26B—C26—H26C	109.5	C7—N3—H3A	115 (3)
N6—C27—C28	121.2 (3)	O6—N4—O5	118.7 (3)
N6—C27—C32	122.2 (3)	O6—N4—C30	121.2 (3)
C28—C27—C32	116.5 (3)	O5—N4—C30	120.0 (4)
C29—C28—C27	121.9 (3)	O7—N5—O8	122.5 (3)
C29—C28—H28	119.1	O7—N5—C32	120.0 (4)
C27—C28—H28	119.1	O8—N5—C32	117.2 (3)
C30—C29—C28	118.1 (3)	C27—N6—C33	125.6 (3)
C30—C29—H29	120.9	C27—N6—H6	117 (3)
C28—C29—H29	120.9	C33—N6—H6	117 (3)

### Hydrogen-bond geometry (Å, °)

**Table:**

*D*—H···*A*	*D*—H	H···*A*	*D*···*A*	*D*—H···*A*
N3—H3A···O4	0.86 (5)	1.95 (5)	2.650 (4)	137 (4)
N6—H6···O8	0.86 (5)	1.99 (5)	2.643 (4)	132 (4)
C10—H10A···O5^i^	0.97	2.24	3.078 (5)	144
C23—H23B···O5^i^	0.96	2.46	3.242 (5)	139
C41—H41···O8^ii^	0.93	2.55	3.435 (5)	159

Symmetry codes: (i) −*x*+1, *y*+1/2, −*z*+2; (ii) −*x*+2, *y*−1/2, −*z*.
